# Alterations in Hepatic *FGF21*, Co-Regulated Genes, and Upstream Metabolic Genes in Response to Nutrition, Ketosis and Inflammation in Peripartal Holstein Cows

**DOI:** 10.1371/journal.pone.0139963

**Published:** 2015-10-09

**Authors:** Haji Akbar, Fernanda Batistel, James K. Drackley, Juan J. Loor

**Affiliations:** Department of Animal Sciences and Division of Nutritional Sciences, University of Illinois, Urbana, Illinois, United States of America; University of Louisville School of Medicine, UNITED STATES

## Abstract

In rodents, fibroblast growth factor 21 (*FGF21*) has emerged as a key metabolic regulator produced by liver. To gather preliminary data on the potential importance of *FGF1*, co-regulated genes, and upstream metabolic genes, we examined the hepatic mRNA expression in response to nutrition and inflammation in dairy cows. In experiment 1, induction of ketosis through feed restriction on d 5 postpartum upregulated *FGF21*, its co-receptor *KLB*, and *PPARA* but only elicited a numerical increase in serum FGF21 concentration. In experiment 2, cows in control (CON) or receiving 50 g/d of L-carnitine (C50) from -14 through 21 d had increased *FGF21*, *PPARA*, and *NFIL3* on d 10 compared with d 2 postpartum. In contrast, compared with CON and C50, 100 g/d L-carnitine (C100) resulted in lower *FGF21*, *KLB*, *ANGPTL4*, and *ARNTL* expression on d 10. In experiment 3, cows were fed during the dry period either a higher-energy (OVE; 1.62 Mcal/kg DM) or lower-energy (CON; 1.34 Mcal/kg DM) diet and received 0 (OVE:N, CON:N) or 200 μg of LPS (OVE:Y, CON:Y) into the mammary gland at d 7 postpartum. For *FGF21* mRNA expression in CON, the LPS challenge (CON:Y) prevented a decrease in expression between d 7 and 14 postpartum such that cows in CON:N had a 4-fold lower expression on d 14 compared with d 7. The inflammatory stimulus induced by LPS in CON:Y resulted in upregulation of *PPARA* on d 14 to a similar level as cows in OVE:N. In OVE:Y, expression of *PPARA* was lower than CON:N on d 7 and remained unchanged on d 14. On d 7, LPS led to a 4-fold greater serum FGF21 only in OVE but not in CON cows. In fact, OVE:Y reached the same serum FGF21 concentration as CON:N, suggesting a carryover effect of dietary energy level on signaling mechanisms within liver. Overall, results indicate that nutrition, ketosis, and inflammation during the peripartal period can alter hepatic *FGF21*, co-regulated genes, and upstream metabolic genes to various extents. The functional outcome of these changes merits further study, and in particular the mechanisms regulating transcription in response to changes in energy balance and feed intake.

## Introduction

Fibroblast growth factor 21 (*FGF21*) is a novel metabolic regulator of the FGF family that is produced by the liver and in rodents has an important role in the regulation of glucose and lipid metabolism [1, 2]. In non-ruminants, the induction of mRNA expression of hepatic *FGF21* stimulates glucose uptake in adipocytes and skeletal muscle, thus, improving insulin sensitivity and reducing serum triacylglycerol (TAG) concentrations [3, 4]. Hepatic *FGF21* also plays a role in regulation of hepatic oxidation of fatty acids and gluconeogenesis in response to fasting and during consumption of high-fat diets [5]. The physiological importance of *FGF21* has been partly demonstrated using *FGF21*-null mice fed a ketogenic diet, which led to higher rates of lypolysis during fasting and greater deposition of liver TAG [6, 7]. Schoenberg et al. [8] working with periparturient cows reported that the onset of negative energy balance (NEB) after calving was associated with increased plasma FGF21 concentration and greater *FGF21* mRNA expression in liver.

Carnitine has an important role in various metabolic functions including mitochondrial long-chain fatty acid (LCFA) oxidation, and has been shown to dramatically decrease or prevent liver lipid accumulation in laboratory animals [9, 10] and dairy cows [11]. Recently, Schlegel et al. [12] working with periparturient cows observed a positive correlation between *FGF21* mRNA expression and genes involved in carnitine synthesis. Furthermore, in rodents the onset of infection, inflammation, trauma, and malignancy induces the acute-phase response (APR), which leads to a decrease in hepatic oxidation of fatty acids and ketogenesis [13,14]. Feingold et al. [15] proposed that FGF21 is a positive APR protein that could help protect animals from the toxic effects of LPS and sepsis.

The above data led us to hypothesize that FGF21 has a central role in the adaptations to NEB, ketosis, carnitine supplementation, and inflammatory challenge in peripartal dairy cows. Thus, the aim of the present study was to develop a better understanding of the role of hepatic *FGF21*, co-regulated genes, and upstream metabolic genes related to hepatic metabolism. To achieve this aim we used liver and serum samples from previous experiments dealing with early postpartal ketosis[16], peripartal dietary L-carnitine supplementation[11], and prepartal level of dietary energy and postpartal intramammary LPS challenge [17].

## Materials and Methods

### Experimental Design and Treatments

The present study was performed using samples from three different experiments, i.e. early postpartal ketosis[16, 18], peripartal dietary L-carnitine supplementation[11], and prepartal dietary energy level and postpartal inflammatory challenge [17]. All these experiments were performed at the University of Illinois Dairy Research Center under protocols approved by the Institutional Animal Care and Use Committee (IACUC) of the University of Illinois.

#### Early Postpartal Ketosis

The details of the animal management and sample collection were presented earlier [16, 18]. Briefly, all Holstein cows were housed in individual tie-stalls, were fed twice daily at ~1000 and 1500 h, and had unlimited access to fresh water. On d 5 postpartum, cows were randomly assigned to control (n = 7) or ketosis-induction (n = 7) based on a thorough physical examination on d 4 postpartum. Cows in the ketosis-induction group were fed at 50% of d 4 intake from d 5 until d 14 postpartum or until they developed signs of clinical ketosis (anorexia, ataxia, or abnormal behavior) while the control group were fed ad libitum throughout the treatment period. The cows with ketosis had higher (*P* < 0.05) concentrations of serum NEFA, BHBA but lower glucose, as well as greater total lipid and TAG in liver than did control cows [16]. A single liver biopsy for gene expression analysis was performed prior to the morning meal between d 9 and 14 (ketosis induction) or d 14 postpartum (control). The serum samples for FGF21 analysis were from the same day of the biopsy. Energy balance of the cows at the time of liver biopsy was greater (*P* < 0.05) for the control (93% of estimated requirements) compared with the ketotic cows (53% of estimated requirements) [18].

#### Dietary Carnitine Supplementation

Detailed information of the animal management and sample collection was reported by[11]. Briefly, all Holstein cows were housed in individual tie-stalls, were fed twice daily at ~1000 and 1500 h, and had unlimited access to fresh water. Cows were assigned to treatments at d −25 relative to expected calving date and remained on experiment until d 56. Treatments were four amounts of supplemental dietary L-carnitine (L-carnitine; Lonza, Inc., Allendale, NJ): control (CON, 0 g/d of L-carnitine; n = 14); low carnitine (6 g/d; n = 11); medium carnitine (C50, 50 g/d; n = 12); and high carnitine (C100, 100 g/d; n = 12). Carnitine supplementation began on d −14 relative to expected calving and continued until d 21. Liver biopsies harvested at d 2 and 10 postpartum before the morning meal were used for gene expression analysis. Blood serum samples from this study were not available for FGF21 determination. Hepatic concentration of free carnitine on d 2 and 10 was greater (*P* < 0.05) than controls with C50 and C100 [11]. Furthermore, at d 2 and 10 higher (*P* < 0.05) total lipid and TAG concentrations were observed in liver from CON cows compared with C50 and C100 [11]. Therefore, liver tissue from d 2 and 10 from CON (n = 6), C50 (n = 6), and C100 (n = 6) were used for gene expression analysis. Energy balance of the cows at the time of liver biopsy was greater (*P* < 0.05) in CON (87% of estimated requirements) and C50 (85% of estimated requirements) compared with C100 (63% of estimated requirements) [11].

#### Prepartal Dietary Energy and Postpartal Intramammary LPS Challenge

Detailed information of the animal management and sample collection was presented by[17]. Briefly, all Holstein cows were housed in individual tie-stalls, were fed twice daily at ~1000 and 1500 h, and had unlimited access to fresh water. Cows were assigned randomly (n = 20 per diet) to a lower-energy diet (CON, high-fiber; 1.34 Mcal/kg DM), which was fed ad libitum to provide approximately 100% of calculated NE_L_ requirements, or were fed a diet to provide at least 150% of calculated NE_L_ requirements (OVE, overfed group; 1.62 Mcal/kg DM) during the entire 45 days of dry period [19]. After parturition, cows were moved to a tie-stall barn, fed a common lactation diet (NE_L_ = 1.69 Mcal/kg DM), and milked twice daily (0400 and 1600 h).

At d 7 postpartum, each group (i.e. CON and OVE) were further divided into two additional groups (total of 4 groups) based on whether they received an intramammary *E*. *coli* LPS challenge (200 μg, strain 0111:B4, cat. # L2630, Sigma Aldrich, St. Louis, MO) or not, i.e. 0 (OVE:N, CON:N) or 200 μg LPS (OVE:Y, CON:Y). Liver tissue harvested on d 7 (2.5 h post-LPS for OVE:Y, CON:Y) and d 14 postpartum (n = 6/dietary group) before the morning meal was used for gene expression analysis. Blood sampled from the coccygeal vein or artery on d 7 (prior to LPS and liver biopsy) and 14 relative to parturition was used for FGF21 determination. Energy balance of the cows at the time of liver biopsy was greater (*P* < 0.05) in CON (78% of estimated requirements) compared with OVE (65% of estimated requirements) [17].

### RNA Extraction, Primer Design, and qPCR Analysis

The complete procedures for RNA extraction and qPCR analysis have been published previously[20,21]. Briefly, approximately 0.2 to 0.3 g of liver tissue was homogenized in 1 to 2 mL ice-cold TRIzol reagent (Invitrogen, Carlsbad, CA, Cat. No. 15596–026) and RNA extraction was performed as described previously[22]. Concentration of RNA was measured using a NanoDrop ND-1000 spectrophotometer (NanoDrop Technologies, Wilmington, DE), while the RNA quality was assessed using a 2100 Bioanalyzer (Agilent Technologies Inc., Santa Clara, CA). The average RNA integrity number value of all samples used was 8 ± 0.4. Total RNA was purified with the RNeasy Mini Kit and residual DNA removed using the RNase-Free DNase Set (Qiagen, Valencia, CA). The extracted and cleaned RNA was re-suspended in RNase free water (Qiagen, Cat No. 74104) and stored at −80°C until qPCR analysis.

The evaluation of direct links among nutrition, ketosis and inflammation were evaluated through the assessment of genes associated with fatty acid oxidation (carnitine palmitoyltransferase 1A, *CPT1A*; peroxisome proliferator-activated receptor alpha, *PPARA*), *FGF21* signaling (klotho beta, *KLB*), and the hepatokine angiopoietin-like 4 (*ANGPTL4*). In addition, recent data from rodent work provided evidence that the *BMAL1* (aryl hydrocarbon receptor nuclear translocator-like, *ARNTL*)*-CLOCK* complex activates the rodent *FGF21* promoter, whereas another circadian gene (nuclear factor, interleukin 3 regulated, *NFIL3*) suppresses it[23, 24]. Furthermore, the downregulation of *FGF21* with insulin in non-ruminants is mediated through *NFIL3*[23]. Therefore, the expression of selected genes associated with circadian rhythms (*ARNTL;* clock circadian regulator, *CLOCK*; *NFIL3*) and insulin signaling (v-akt murine thymoma viral oncogene homolog 1, *AKT1*) also were investigated. The final data were normalized using the geometric mean (stability = 0.20) [25] of ubiquitously-expressed transcript (*UXT*), glyceraldehyde-3-phosphate dehydrogenase (*GAPDH*), and ribosomal protein S9 (*RPS9*). Details of the primers and sequences are presented in supplementary **Tables A-D in the S1 File**.

### Serum Concentration of FGF21

Because FGF21 is a secreted protein, ELISA was performed to determine the serum concentration of FGF21 using a Mouse and Rat FGF-21 ELISA (BioVendor, Laboratorni medicina a.s., Brno, Czech Republic, Cat. No. RD291108200R). The antibody provided with the kit reportedly has cross reactivity with the bovine FGF21 protein, which we previously confirmed[20]. However, it should be kept in mind that the primary antibody also cross-reacts with other bovine proteins. Thus, the data generated should be regarded as qualitative than quantitative. All the procedures were performed according to the manufacturer’s protocol. The minimal detectable concentration of FGF21 with this assay estimated by the manufacturer is 18.4 pg/mL. The lowest concentration detected in cow serum in the present study was 48.88 pg/mL. The intra- and inter-assay coefficients of variation were less than 9.0% and less than 8.0%, respectively.

### Statistical Analysis

The MIXED procedure of SAS (version 9.1; SAS Institute Inc., Cary, NC) was used for statistical analysis. In experiment 1, diet (control and ketosis) was included as fixed effect. In experiment 2, the model considered as fixed effects diet (CON, C50, and C100), d (2 and 10) and the interaction between diet and day. A repeated measures analysis was performed using the AR(1) covariate structure. In experiment 3, the model included as fixed effects diet (CON and OVE), LPS (No and Yes), d (7 and 14) and all possible interactions. Data were normalized by logarithmic transformation prior to statistical analysis. All means were compared using the PDIFF statement of SAS. Gene expression data reported in tables were back-transformed after statistical analysis. The 95% confidence intervals were calculated with logarithmic transformed data using a modified version of the Cox method as suggested by Olsson [26].

## Results

### Hepatic Gene Expression

#### Early Postpartal Ketosis

A greater *FGF21* (*P* < 0.01) and *KLB* (*P* = 0.02) mRNA expression was detected in ketotic cows compared with control cows (**Table 1**). No significant change (*P* > 0.05) was observed between control and ketotic cows for the mRNA expression of *ARNTL*, *CLOCK*, *NFIL3* and *AKT1*. The mRNA expression of *CPT1A* and *PPARA* was not measured in this study because they were reported by Loor et al.[16].

**Table 1:** Hepatic mRNA expression of *FGF21*, FGF21 binding, and genes associated with circadian rhythms and insulin signaling in control cows (n = 7) and cows induced to develop ketosis (n = 7) by undernutrition after calving. Liver biopsy was performed at d 9 to 14 (ketosis induction) or 14 d postpartum (control) before the morning meal. The 95% confidence interval (CI) is reported.

Gene	Treatment	Relative expression^1^	CI	*P* value
*FGF21*	Control	3.26	1.33 to 7.99	<0.01
	Ketotic	133.4	94.61 to 188.20	
*KLB*	Control	7.97	5.66 to 11.24	0.02
	Ketotic	25.5	18.08 to 35.89	
*ARNTL*	Control	1.48	1.17 to 1.87	0.26
	Ketotic	2.16	1.71 to 2.72	
*CLOCK*	Control	0.37	0.29 to 0.47	0.20
	Ketotic	0.57	0.40 to 0.65	
*NFIL3*	Control	0.55	0.43 to 0.70	0.39
	Ketotic	0.74	0.58 to 0.93	
*AKT1*	Control	0.42	0.37 to 0.49	0.86
	Ketotic	0.44	0.38 to 0.50	

^1^Calculated after normalization with the geometric mean of *UXT*, *GAPDH* and *RPS9* (see materials and methods).

#### Dietary L-Carnitine Supplementation

There was an interaction (diet × day; *P* < 0.05; **Table 2**) for all genes except *AKT1* and a tendency for interaction (*P* = 0.06) for *CLOCK*. The mRNA expression of *FGF21*, *CPT1A*, and *ARNTL* was not affected (*P* > 0.05) by diet or time, but an interaction occurred (diet × day; *P* < 0.05). No main effects of carnitine (*P* > 0.05) were observed for *AKT1*. Supplemental carnitine did not affect *FGF21* at d 2, while at d 10 feeding C100 resulted in a marked decrease in *FGF21* compared with control. No main effect of carnitine or time were observed for *CPT1A* expression; however, feeding C50 increased *CPT1A* expression at d 2 compared with d 10 (diet × day; *P* < 0.05). At d 2, no change was observed in the expression of *ARNTL* between the treatments, whereas at d 10 feeding C100 decreased the expression of *ARNTL* compared with control and C50. Furthermore, between d 2 and 10, *ARNTL* expression was lower in cows fed C100.

**Table 2:** Hepatic mRNA expression of *FGF21*, FGF21 binding, and genes associated with circadian rhythms and insulin signaling in cows fed control (0 L-carnitine; n = 6) and dietary L-carnitine at a rate of 50 g/d (C50; n = 6) or 100 g/d (C100; n = 6) from d −14 through 21 around parturition. Liver biopsies harvested at d 2 and 10 postpartum before the morning meal. The 95% confidence interval is reported in parentheses.

Gene	Day	Treatment			*P* value		
		CON	C50	C100	Diet (T)	Day (D)	T × D
*FGF21*	2	0.89 (0.52 to 1.22)	0.32 (0.19 to 0.53)	0.62* (0.40 to 0.74)	0.21	0.85	0.03
	10	1.34^A^ (0.80 to 1.51)	0.51^A^ ^B^ (0.30 to 0.84)	0.23^B^ * (0.15 to 0.34)			
*PPARA*	2	1.16 (1.04 to 1.29)	1.06* (0.97 to 1.18)	1.11 (0.99 to 1.24)	0.08	0.68	0.02
	10	1.23^A^ (1.14 to 1.37)	1.33^A^ * (1.23 to 1.50)	1.04^B^ (0.93 to 1.10)			
*KLB*	2	1.08 (0.95 to 1.23)	0.93 (0.82 to 1.05)	1.06* (0.92 to 1.21)	<0.01	0.46	<0.01
	10	1.06^A^ (0.94 to 1.20)	1.02^A^ (0.90 to 1.16)	0.69^B^ * (0.60 to 0.80)			
*CPT1A*	2	1.01 (0.90 to 1.12)	0.91* (0.82 to 1.02)	1.06 (0.94 to 1.19)	0.81	0.39	0.05
	10	1.13 (1.07 to 1.25)	1.11* (1.04 to 1.24)	0.90 (0.80 to 1.02)			
*ANGPTL4*	2	0.73^A^ * (0.63 to 0.85)	1.23^B^ (1.06 to 1.42)	1.26^B^ * (1.07 to 1.48)	0.23	<0.01	<0.01
	10	1.05^A^ * (0.91 to 1.21)	1.01^A^ (0.87 to 1.16)	0.53^B^ * (0.45 to 0.62)			
*ARNTL*	2	0.37 (0.28 to 0.48)	0.24 (0.19 to 0.32)	0.30* (0.23 to 0.39)	0.37	0.41	<0.05
	10	0.33^A^ (0.25 to 0.42)	0.33^A^ (0.26 to 0.42)	0.17^B^ * (0.13 to 0.22)			
*CLOCK*	2	0.73^A^ (0.45 to 1.19)	0.08^B^ * (0.05 to 0.14)	0.93^A^ (0.53 to 1.60)	0.06	0.02	0.06
	10	0.76 (0.47 to 1.23)	0.36* (0.24 to 0.62)	1.05 (0.59 to 1.82)			
*NFIL3*	2	0.98^A^ * (0.88 to 1.10)	1.34^B^ * (1.20 to 1.50)	1.21^A^ ^B^ (1.06 to 1.36)	<0.01	0.14	0.03
	10	1.35^A^ ^B^ * (1.22 to 1.56)	1.72^B^ * (1.54 to 1.92)	1.21^A^ (1.07 to 1.36)			
*AKT1*	2	0.12 (0.10 to 0.14)	0.13 (0.11 to 0.15)	0.16 (0.14 to 0.18)	0.33	0.49	0.26
	10	0.13 (0.12 to 0.15)	0.14 (0.13 to 0.16)	0.15 (0.13 to 0.17)			

^1^Calculated after normalization with the geometric mean of *UXT*, *GAPDH* and *RPS9* (see materials and methods).

*Means within the treatment group differ (*P <* 0.05) between d 2 and 10.

^ABC^Within time point (d 2 and 10), treatment means (CON, C50 and C100) without a common superscript differ (*P* < 0.05).

There was an interaction (diet × day; *P* < 0.05; **Table 2**) for *PPARA*, *KLB*, and *NFIL3*. The mRNA expression of *PPARA*, *KLB*, and *NFIL3* was affected by diet (*P* < 0.01); however, no effect of time (*P* > 0.05) was observed for these genes. The mRNA expression of *PPARA* and *KLB* were not affected by diets at d 2, whereas at d 10 the C100 decreased their expression. Feeding C50 increased expression of *PPARA* and C100 increased expression of *KLB*. Furthermore, at d 10 mRNA expression of *PPARA* and *KLB* was lower with C100 in comparison with control and C50. At d 2, the mRNA expression of *NFIL3* was increased with feeding C50 (*P* < 0.05) in comparison with control; while at d 10 feeding C100 decreased (*P* < 0.05) *NFIL3* expression compared with C50.

The mRNA expression of *ANGPTL4* decreased (diet × day, *P* < 0.05; **Table 2**) from d 2 to 10 with the highest dose of carnitine (C100) supplementation, whereas an increase was observed in controls between d 2 and 10 relative to parturition (*P* < 0.01). However, no significant change was observed in cows fed C50 between d 2 and 10 relative to parturition. Overall, at d 2, the control had lower mRNA expression of *ANGPTL4*, but at d 10 feeding C100 result in a marked decrease. A tendency for a main effect of diet (*P* = 0.06) and an interaction (*P* = 0.06) were observed for *CLOCK*. At d 2, C50 decreased *CLOCK* mRNA expression in comparison with control and C100; however, C50 did not induce differences (*P* > 0.05) compared with other treatments at d 10.

#### Prepartal Energy and Postpartal Intramammary LPS Challenge

Interactions among diet, time, and LPS (*P* < 0.05) were observed for *FGF21*, *PPARA*, *NFIL3*, and *CLOCK* (**Table 3**). A day × LPS interaction (*P* < 0.05) was observed for *ANGPTL*, *NFIL3*, *CLOCK*, *ARNTL*, and *AKT1*. Except for *FGF21* (*P* = 0.07), no other gene had an interaction between diet and LPS. A diet × day (*P* < 0.05) interaction was only observed for *AKT1*.

**Table 3:** Hepatic mRNA expression of *FGF21*, FGF21 binding, and genes associated with circadian rhythms and insulin signaling in cows overfed energy (OVE:N; n = 6) or fed to meet energy requirements (CON:N; n = 6) during the entire dry period, and receiving an intramammary LPS challenge at d 7 postpartum (CON:Y, OVE:Y). Liver tissue harvested at 2.5 h post-LPS on d 7 and 14 postpartum before the morning meal. The 95% confidence interval is reported in parenthesis.

Gene	Day	Treatment				*P* value						
		OVE:N	OVE:Y	CON:N	CON:Y	Diet (T)	Day (D)	LPS (L)	T × D	T × L	D × L	T × D × L
*FGF21*	7	0.81^A^ ^B^ (0.49 to 1.33)	1.27^A^ * (1.08 to 1.47)	0.25^B^ * (0.16 to 0.52)	0.82^A^ ^B^ (0.51 to 1.33)	<0.01	0.04	0.07	0.11	0.07	0.56	<0.01
	14	0.87^A^ (0.53 to 1.42)	0.55^A^ * (0.34 to 0.84)	0.06^B^ * (0.03 to 0.10)	0.66^A^ (0.41 to 1.07)							
*PPARA*	7	0.77^A^ * (0.68 to 0.88)	0.81^A^ (0.72 to 0.92)	1.13^B^ (0.97 to 1.32)	0.86^A^ ^B^ * (0.76 to 0.98)	0.06	<0.01	0.42	0.43	0.68	0.82	<0.01
	14	1.01^A^ ^B^ * (0.90 to 1.15)	0.87^A^ (0.77 to 0.98)	1.26^B^ (1.08 to 1.47)	1.20^B^ * (1.06 to 1.37)							
*CPT1A*	7	1.10 (1.01 to 1.19)	1.05 (0.97 to 1.14)	0.89 (0.83 to 1.06)	1.01 (0.94 to 1.10)	0.16	0.53	0.85	0.74	0.16	0.27	0.27
	14	1.14 (1.04 to 1.24)	0.97 (0.89 to 1.09)	0.88 (0.80 to 1.05)	1.01 (0.93 to 1.09)							
*ANGPTL4*	7	1.27 (0.99 to 1.80)	2.19 (1.72 to 2.80)	0.51 (0.38 to 0.70)	2.27 (1.77 to 2.90)	0.06	<0.01	0.21	0.66	0.16	<0.01	0.17
	14	0.96 (0.65 to 1.22)	0.52 (0.41 to 0.67)	0.46 (0.33 to 0.72)	0.39 (0.31 to 0.50)							
*KLB*	7	1.13 (0.65 to 1.47)	0.24 (0.14 to 0.41)	1.06 (0.54 to 1.54)	0.15 (0.09 to 0.24)	0.82	0.32	<0.01	0.32	0.77	0.30	0.83
	14	1.03 (0.59 to 1.35)	0.35 (0.10 to 0.26)	1.17 (0.60 to 1.75)	0.13 (0.08 to 0.23)							
*ARNTL*	7	1.37 (1.04 to 1.51)	0.03 (0.02 to 0.05)	0.95 (0.70 to 1.26)	0.02 (0.01 to 0.04)	0.71	0.67	<0.01	0.19	0.73	<0.01	0.26
	14	0.83 (0.51 to 1.10)	0.03 (0.02 to 0.05)	0.60 (0.44 to 0.87)	0.05 (0.03 to 0.08)							
*CLOCK*	7	0.95^A^ (0.72 to 1.24)	0.14^B^ * (0.08 to 0.22)	1.04^A^ (0.82 to 1.37)	0.11^B^ (0.07 to 0.18)	0.63	0.10	<0.01	0.20	0.75	0.02	0.01
	14	0.95^A^ (0.70 to 1.29)	0.04^B^ * (0.02 to 0.06)	1.02^A^ (0.78 to 1.32)	0.11^B^ (0.06 to 0.19)							
*NFIL3*	7	1.09^A^ (0.93 to 1.29)	4.03^B^ * (3.42 to 4.71)	1.24^A^ * (1.15 to 1.38)	4.69^B^ * (3.99 to 5.14)	0.25	<0.01	0.38	0.10	0.26	<0.01	0.02
	14	1.08^A^ (0.92 to 1.28)	0.47^B^ * (0.40 to 0.55)	1.69^C^ * (1.43 to 2.00)	0.58^B^ * (0.49 to 0.68)							
*AKT1*	7	1.34 (1.17 to 1.53)	0.43 (0.37 to 0.49)	1.23 (1.05 to 1.44)	0.39 (0.33 to 0.44)	0.77	0.07	<0.01	0.02	0.69	<0.01	0.44
	14	1.27 (1.11 to 1.45)	0.28 (0.25 to 0.32)	1.65 (1.40 to 1.93)	0.30 (0.27 to 0.35)							

^1^Calculated after normalization with the geometric mean of *UXT*, *GAPDH* and *RPS9* (see materials and methods).

*Means within the treatment group (OVE:N, OVE:Y, CON:N and CON:Y) differ (*P* < 0.05) between d 7 and 14.

^ABC^Within time point (d 7 and 14), treatment means (OVE:N, OVE:Y, CON:N and CON:Y) without a common superscript differ (*P* < 0.05).

LPS challenge tended (diet × LPS, *P* = 0.07) to increase *FGF21* mRNA expression at d 7 in OVE:Y and CON:Y compared with the respective controls (**Table 3**). However, OVE:Y and CON:N resulted in a marked decrease (diet × day × LPS, *P* < 0.01) in mRNA expression from d 7 to 14 and a nadir in expression was observed in CON:N. The mRNA expression of *PPARA* was higher (diet × day × LPS, *P <* 0.05) at d 14 in CON:N and CON:Y groups, whereas no significant change (*P >* 0.05) was detected for the mRNA expression of *CPT1A*. The mRNA expression of *ANGPTL4* was greater (day, *P <* 0.05) at d 7, and in OVE:N compared with CON:N (diet, *P =* 0.06). Furthermore, LPS increased the mRNA expression of *ANGPTL4* in both OVE:Y and CON:Y and, similar to controls (OVE:N and CON:N), mRNA expression decreased (LPS × diet, *P <* 0.05) on d 14. Regardless of prepartal dietary energy, LPS markedly decreased (LPS, *P <* 0.05) *KLB* mRNA expression.

The mRNA expression of genes associated with the circadian rhythm was significantly affected by the interaction of diet, day, and LPS challenge (*P* < 0.05; **Table 3**); *NFIL3* increased while a decrease was observed for *ARNTL* and *CLOCK* mRNA expression in cows receiving the LPS challenge (OVE:Y, CON:Y) at d 7. Furthermore, results revealed an increase (diet × LPS, *P* < 0.05) in *NFIL3* mRNA expression due to LPS challenge (OVE:Y, CON:Y) at d 7; however, that was followed by a decrease at d 14. The mRNA expression of *ARNTL* was greater (day, *P* < 0.05) in OVE:N at d 7 while it decreased at d 14. The LPS challenge decreased (diet × LPS, *P* < 0.05) its expression in both groups, but CON:Y increased *ARNTL* between d 7 and d 14. Furthermore, the LPS treatment decreased (*P* < 0.05) the mRNA expression of *CLOCK* irrespective of diet (day × LPS, *P* > 0.05), and a further decrease (diet × LPS, *P* < 0.05) was detected in OVE:Y at d 14. The mRNA expression of *AKT1* increased (diet × LPS, *P* > 0.05) at d 14 in CON:N compared with other groups; however, a decrease was observed in the LPS-challenged groups.

### Serum Concentration of FGF21

**Fig 1:**
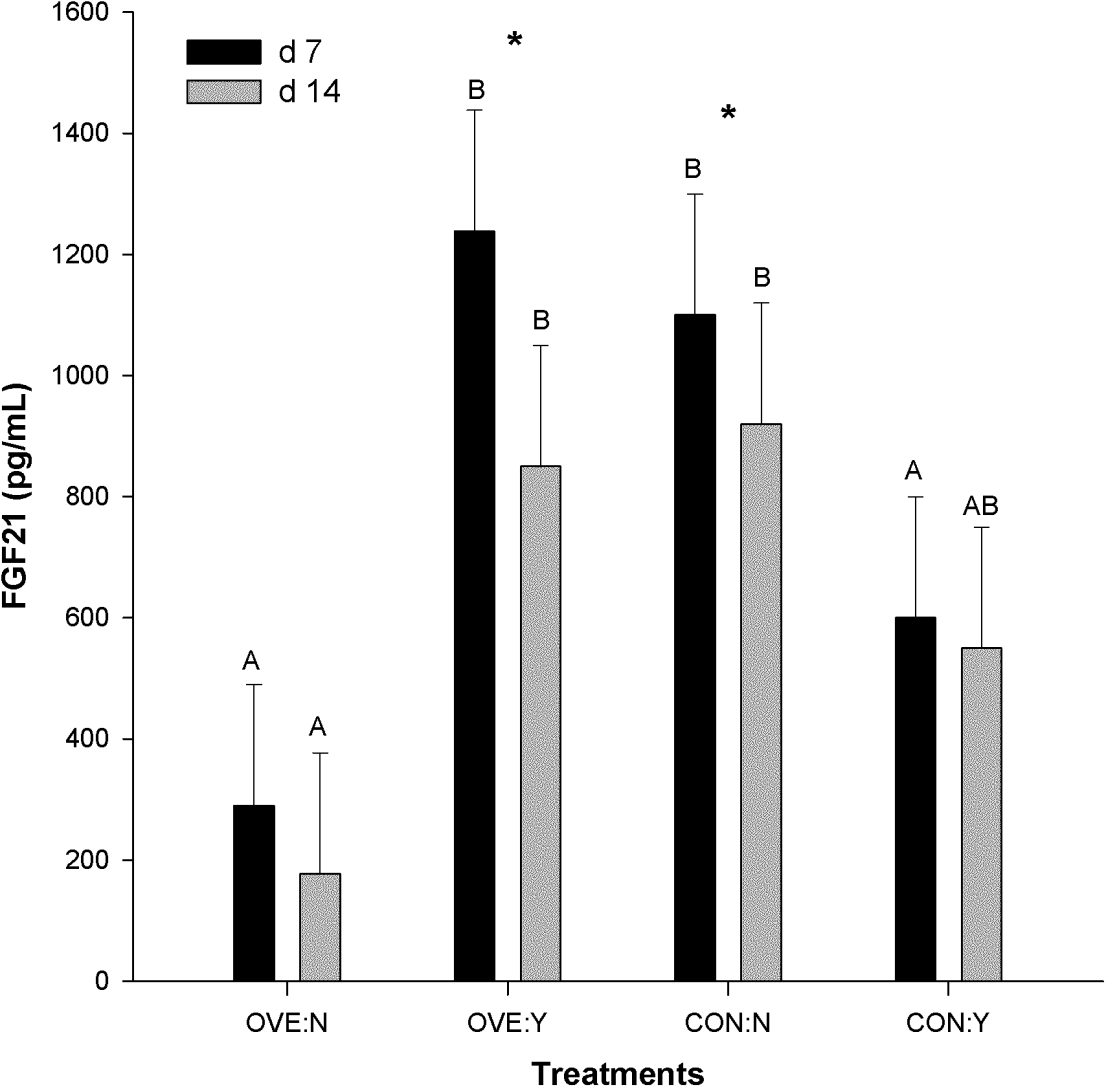
Serum concentration of FGF21 in cows overfed energy (OVE:N, n = 6) or fed to meet energy requirements (CON:N, n = 6) during the entire dry period, and receiving an intramammary LPS challenge at d 7 postpartum (OVE:Y, CON:Y). The error bars represent SEM. The serum contraction of FGF21 tended to have an interaction among diet, time, and LPS (*P* = 0.08). No main effected of diet (*P* = 0.48) or LPS (*P* = 0.37) was observed, but the interaction diet × LPS was significant (*P* < 0.01). The statistical effects of diet, LPS, days are indicated: *Significant difference at 7 and 14 d postpartum within the same treatment groups; ^A,B^Significant difference among treatments (diet with or without LPS) at d 7 or 14 postpartum.

In the absence of serum samples from the dietary carnitine experiment [11], we only measured the blood serum concentration of FGF21 from the ketosis [16]and prepartal higher-energy diet and LPS challenge studies [17]. Although we observed greater mRNA expression of *FGF21* in the liver of ketotic cows, the serum concentration of FGF21 was only numerically different (*P* = 0.25) between diets (**Fig 1;** 1370 ± 279 vs. 905 ± 275 pg/mL).

The serum contraction of FGF21 tended (*P* = 0.08) to have an interaction among diet, time, and LPS. No main effected of diet (*P* = 0.48) or LPS (*P* = 0.37) was observed, but the interaction diet × LPS was significant (*P* < 0.01). Intramammary LPS challenge increased (*P* < 0.05) the blood concentration of *FGF21* in OVE:Y but decreased it (diet × LPS, *P* < 0.05) in CON:Y (**Fig 1**). Furthermore, OVE:Y and CON:N decreased serum FGF21 concentration between d 7 and 14 (diet × LPS, *P* < 0.05), whereas OVE:N and CON:Y only had a numerical decrease.

## Discussion

### Ketosis Induces the mRNA Expression of *FGF21*

Early lactation in dairy cows is a nutritionally precarious period owing to a combination of increases in energy requeriments for milk production and decreases in voluntary feed intake[27]. During periods of NEB, high-producing cows become more susceptible to developing ketosis, leading to further increases in NEFA, BHBA, hepatic TAG concentration, hepatic mRNA expression of *PPARA* and angiopoietin-like 4 (*ANGPTL4*, a hepatokine), and other genes associated with ketogenesis and gluconeogenesis[16]. The 41-fold increase in *FGF21* mRNA expression in ketotic cows agrees with previous observations in cows with NEB [8]. Work in rodents revealed that plasma FGF21 increases during fasting and it plays a critical role in metabolic fuel homeostasis during ketosis [28]. It was suggested that increased *FGF21* in obese humans may protect against chronic exposure to high concentrations of NEFA, which are toxic in muscle, pancreas, and liver [29]. Schoenberg et al. [8] observed a substantial individual variation in FGF21 serum concentration among cows, particularly at the time of calving and very early in lactation. Therefore, the lack of significant difference in FGF21 serum concentration in the present study can be partly attributed to small sampling size and the high variation among cows. Overall, a period of energy deficit in dairy cows is positively correlated with *FGF21* mRNA expression in liver, similar to what is observed in food-deprived rodents and humans [5, 30]. However, it cannot be discerned from our data if the actual reduction of feed intake or the onset of ketosis per se caused the change in *FGF21* expression.

The somatotropic axis controls many aspects of growth and lactation; calving and NEB are associated with uncoupling of the somatotropic axis [31]. Calving increases blood concentration of growth hormone (GH), which further augments blood NEFA during NEB [32–34]. In non-ruminants, the NEFA can trigger hepatic *FGF21* and *ANGPTL4* upregulation and prodution through the activation of *PPARA* [35,36]. In turn, activation of *PPARA* and *FGF21* are correlated with an increase in the hepatic protein concentration of CPT1A and HMGCS2 via a posttranscriptional mechanism without changes in mRNA[1, 28]. The lack of change in CPT1 activity in hepatic mitochondria [37] and in the mRNA expression of *CPT1A* with ketosis [16] suggests that CPT1A might not have been affected by ketosis even though FGF21 mRNA expression increased and serum contration was numerically higher.

Yu et al. [38] also observed in beef cattle that GH enhances hepatic *FGF21* gene transcription and that this stimulation is mediated, at least in part, by the transcription factor STAT5. They suggested that *FGF21* inhibits the JAK-2/STAT5 signaling through a negative loop involving the binding of GH to the hepatic GH receptor (GHR) upon which JAK2-STAT5 signalling starts and can regulate target genes such as *FGF21*, *IGF1*, and *SOCS2*. However, the downregulation of *GHR*, *STAT5*, *IGF1*, and *SOCS2* in cows with ketosis in our study [16] does not appear to support the model proposed by Yu et al. [38]. The discrepancy is explained in part by the fact that hepatic *GHR* decreases around parturition in dairy cattle but not in beef cattle [39]. The decrease in hepatic mRNA expression of *STAT5B* and *SOCS2* coincides with the uncoupling of GH/IGF-1 axis [31].

The specificity of secreted FGF21 action is mediated through FGF receptors (FGFR 1–4) in peripheral tissues, but requires the presence of a co-receptor (KLB) in order to elicit a response [40, 41]. The fact that ketosis increased the hepatic mRNA expression of *KLB* in a similar fashion to *FGF21* at the onset of lactation [8] suggests that *FGF21* may exert a paracrine/autocrine action on liver during ketosis. As such, *FGF21* may lead to a decrease in the hepatic mRNA expression of *IGF1* by increasing the mRNA expression of *IGFBP1* [42, 43], which was reported previously [16].

### Dietery Carnitine downregulates the mRNA Expression of *FGF21*

Carlson et al. [11] using the same cows from the present study reported that C50 and C100 resulted in lower liver TAG than control on d 2 and 10, but only feeding C100 led to greater concentration of BHBA compared with control or C50. The lower DMI caused by feeding C100 partly explains the greater BHBA[11]. Although both feeding C50 and C100 increased liver synthesis of acid soluble products (i.e. ketones), the fact that cows fed C100 had lower milk production [11] could partly explain the greater BHBA compared with C50.

During early lactation, adipose tissue lipolysis results in elevated plasma NEFA that can be taken up by liver (~25% of the NEFA flux) to meet its oxidative needs and to produce ketone bodies for extra-hepatic tissues [44]. In non-ruminant liver, hypoinsulinemia due to undernutrition increases NEFA flux into liver where they activate PPARα and forkhead box A2 (FOXA2) resulting in an overall increase in fatty acid oxidation and ketogenesis [45]. Concomitantly, PPARα activation increases *FGF21* and its concentration in the circulation, and appears responsible for matching adipose tissue mobilization to oxidative capacity in liver [8] by stimulating hepatic NEFA use while concurrently decreasing lipolysis [6].

The marked decrease between d 2 and 10 in mRNA expression of *FGF21* and its co-receptor *KLB* in the C100 cows agrees with the decrease in metabolism of palmitate to acid-soluble products[11], i.e. despite the lower DMI due to feeding C100 the rates of LCFA oxidation to CO_2_ did not differ among groups. Furthermore, the greater insulin concentration detected in cows fed C100 compared with controls also could have had a negative effect on LCFA oxidation as in non-ruminants. It is noteworthy that cows fed C50 did not decrease DMI [11] and had an increase in mRNA expression of *CPT1A* and *PPARA* between d 2 and 10, suggesting the existence of a threshold of L-carnitine above which there is feedback inhibition of fatty acid oxidation. Furthermore, those data support a role for PPARα activation in the process of LCFA oxidation.

In mice, evidence indicates that the circadian protein *NFIL3* alters *FGF21* mRNA expression during a circadian cycle and upon food intake [23]. Expression of *FGF21* peaks during the post-absorptive phase, but *NFIL3* is highest during the fed state; insulin increases *NFIL3* mRNA expression and binding to the *FGF21* promoter through AKT activation, indicating that *NFIL3* is an insulin-responsive repressor of *FGF21* mRNA expression [23]. This protein also suppressed the *BMAL1* (*ARNTL*)*-CLOCK*-activated *FGF21* mRNA expression and abolished the PPARα-activated *FGF21* mRNA expression [23]. Despite the lack of change in expression of *AKT1*, the greater blood insulin in C50 compared with controls [11] agrees with the increase in *NFIL3* mRNA expression between d 2 and 10 and the concomitant decrease in mRNA expression of *FGF21*. Together, these data are suggestive that *NFIL3* could regulate FGF21 synthesis in ruminants.

A growing amount of evidence indicates that *FGF21* and *ANGPTL4* are liver-derived biomarkers of energy balance during the peripartal period [20, 22]. Thus, the better energy balance [11] and greater mRNA expression of *ANGPTL4* and *FGF21* observed in the control group indicate that there are “energy-independent” mechanisms that might control mRNA expression of these genes. For example, the higher ketone body concentration with both carnitine treatments could have promoted pancreatic insulin secretion as reported in rats [46]. As such, ketone bodies could have played a role in mediating the downregulation of *FGF21* through *NFIL3* [23]. The fact that control cows had lower blood insulin concentrations offers support for this mechanism.

### Prepartal Energy and LPS Challenge Alter *FGF21* mRNA Expression

Overfeeding dairy cows during the dry period leads to greater BCS before calving, a pronounced decrease in appetite around calving, and as a result more severe NEB [47]. The overfed cows used in this study were in positive energy balance and had greater BCS prepartum than controls, but were in more NEB early postpartum and before the LPS challenge [17]. After the LPS challenge, blood BHBA decreased, NEFA increased, and liver TAG increased only in the OVE:Y group [17]. The observed increase in *FGF21* mRNA expression due to feeding OVE without the LPS challenge was associated with higher NEFA concentration, as reported previously [8]. However, the lack of significant effect on the mRNA expression of *PPARA* due to OVE or LPS suggests the existence of a PPARα-independent mechanism that controls mRNA expression of *FGF21* during inflammation or feeding OVE. The lack of a *PPARA* response to high NEFA in some studies with transition cows [48, 49] supports this idea. Palin and Petit [50] observed no change in hepatic mRNA expression of *PPARA* in response to feeding above 100% of prepartal energy requirements and different sources of fat. Furthermore, Carriquiry et al. [48] speculated that the lack of increase in *PPARA* postpartum in response to diets providing 8% more fat than typically fed to dairy cows and enriched with PUFA could be related with a hepatic inflammatory response induced by prepartal high-fat intake. All these observations suggest the existence of a PPARα-independent mode of action on the regulation of *FGF21* in dairy cows consuming high-prepartal energy and fat levels and afflicted by inflammatory conditions after calving.

In non-ruminants, recent evidence indicates that the circadian protein NFIL3 regulates the hepatic mRNA expression of *FGF21* during a circadian cycle and upon food intake [23]. In ruminants, a recent study provided some evidence for circadian control of FGF21 [51]. The NFIL3 protein suppressed the activation of *FGF21* mRNA expression by *BMAL1* (*ARNTL*)*-CLOCK* and also abolished PPARα activation of *FGF21* expression [23]. In the present study, only the marked upregulation of NFIL3 coupled with the downregulation of CLOCK after LPS in both OVE and CON offer the clearest support for an antagonistic role similar to that in non-ruminants. Whether, the CLOCK response had an effect at the protein expression level for PPARA and ARNTL remains to be determined. The observed increase in *FGF21* mRNA expression (**Table 3**) and serum concentration in OVE:Y (**Fig 1**) support the existence of another regulatory mechanism for *FGF21* mRNA expression under inflammatory conditions.

The marked decrease in hepatic mRNA expression of *STAT5B* and *SOCS2* in OVE:Y between d 7 and 14 relative to parturition [17] confirms the uncoupling of GH/IGF-1 axis [31] and argues against the possibility of GH-mediated activation of *FGF21* via *JAK2/STAT5* signaling [38]. It has been recognized recently in non-ruminants that glucagon increases the hepatic mRNA expression of *FGF21* either via PPARα mediated activation or directly via protein kinase A (PKA) [35, 36]. Calving increases the concentration of glucagon [52] as part of the homeorhetic mechanisms to coordinate lactation. The increase in glucagon around calving [52] and the NEB at d 7 [17] might have led to the activation of PKA. This idea is supported by a recent study in rodents and humans that detected increases in both mRNA expression and secretion of *FGF21* in response to both native glucagon and glucagon receptor (GcgR) agonists [53]. Those data demonstrated that glucagon achieves its long-term effects on energy and lipid and glucose metabolism at least in part via *FGF21*-dependent pathways.

We speculate the existence of a glucagon-mediated activation of *FGF21* via PKA. For example, both intravenous and intramuscular injection of glucagon reduced NEFA and alleviated fatty liver in dairy cows [54, 55]. If FGF21 increased with glucagon injections, then the protein could exert some inhibition of adipose lipolysis as demonstrated in rodents [56]. Therefore, we suggest that glucagon and consequently NEFA can induce hepatic mRNA expression of *FGF21* in order to achieve its lipolysis inhibitory effect. Further studies are required to demonstrate these mechanistic relationships.

The increase in serum concentration of FGF21 with LPS challenge agrees with the greater mRNA expression in the liver, which is the main contributor of blood FGF21 in dairy cows [8]. Glucagon is a potent anti-inflammatory hormone [57], thus, the upregulation of *FGF21* with LPS also might be attributed to an increase in glucagon coupled with a decrease in DMI [17]. As reported in non-ruminants [58], in bovine, PPARδ was suggested to regulate the mRNA expression of *ANGPTL4* and other hepatokines during acute inflammation[59]. Furthermore, in the cows under study the LPS challenge induced the hepatic mRNA expression of *PPARD* and genes associated with inflammation and stress [17]. The parallel increase of *ANGPTL4* mRNA expression with LPS, which has recently been reported as a positive acute-phase protein in mice challenged with LPS [60], supports the speculation that LPS induced the hepatic mRNA expression of both hepatokines *ANGPTL4* and *FGF21* via PPARδ.

Our observations of increased serum FGF21 in response to LPS administration agrees with the increase in serum FGF21 with LPS injection in mouse [15]; however, in contrast with our results, Feingold et al. [15] demonstrated that adipose tissue is a major contributor of serum concentration of FGF21 in LPS-challenged mice. Previous work from our group [61] failed to detect *FGF21* mRNA expression in subcutaneous adipose tissue around parturition, casting doubt on the contribution of this tissue to peripheral FGF21 concentration. Furthermore, Schoenberg et al. [8] observed little or no mRNA expression of *FGF21* in white adipose tissue, skeletal muscle, and mammary gland, suggesting those tissues do not contribute to circulating FGF21. However, as discussed in the previous sections, β-Klotho (*KLB*; *FGF21* co-receptor) determines the target specificity of FGF21 action; down regulation of hepatic *KLB* with LPS suggests an extra-hepatic role of FGF21 during acute inflammation. We speculate that LPS might have induced *FGF21* in adipose tissue to suppress lipolysis in order to reduce the NEFA flow to liver as a way to decrease lipidosis.

Overall, the lack of *FGF21* mRNA expression in subcutaneous adipose tissue around parturition [61] and the observations from Schoenberg et al. [8] support the view that under normal conditions the adipose tissue is not a contributor to blood FGF21. However, in dairy cows it appears that adipose tissue is the second major target tissue after liver. In a survey of 15 tissues that included the mammary gland, the mRNA expression of *KLB* and a subset of interacting FGF receptors was restricted to liver and white adipose tissue and was modestly affected by the transition from late-pregnancy to early lactation in liver but not in adipose [8].

Overall, the data obtained for *FGF21* confirm that this gene is a biomarker of negative energy balance as reported previously [8, 20]. Beyond evidencing the important role of FGF21 in peripartal cow biology, the responses across the three experiments suggest different adaptations in hepatic signaling due to onset of ketosis, nutritional management, and inflammation. For instance, the role of KLB in hepatic adaptations to parturition does not seem to be important unless there are marked changes in NEFA and BHBA production as would occur in clinical ketosis. Acute inflammation proved to be a strong chronic inhibitor of signaling via KLB, the clock network, and potentially the insulin pathway. Furthermore, there seems to be a threshold up to which enhancing LCFA influx into the mitochondria via L-carnitine is effective for allowing liver to achieve higher rates of beta-oxidation at least in part through upregulation of *PPARA* and *CPT1A*. Under those “ideal” conditions the level of intra-hepatic “stress” is diminished, hence, the lower and stable *FGF21*. A novel mechanism of insulin action via *NFIL3* upregulation at “optimal” rates of LCFA oxidation (cows in C50) was surmised, and suggests a degree of insulin sensitivity in liver despite NEB.

## Perspectives

Although the data generated from the studies reported herein provide a preliminary evaluation of the likely roles of liver-derived FGF21 in helping to coordinate the cow’s adaptations to changes in energy balance, onset of ketosis or inflammation, and stimulation of fatty acid oxidation there is a need to conduct more mechanistic studies aimed specifically at deciphering the transcriptional control of *FGF21* in bovine liver. Specifically during the immediate period after parturition when most if not all cows are most susceptible to developing ketosis or an inflammatory disorder. To achieve greater mechanistic understanding, techniques such as ChIP assays could be used. Because there was no bovine antibody specific for FGF21 available at the time these experiments were conducted, there would be a need to replicate them. Furthermore, the differences in feed intake induced by the treatments we studied also confound a precise interpretation of the mechanisms driving the changes in hepatic *FGF21*. Lastly, future work on the regulation of bovine hepatic *FGF21* needs to encompass in vitro studies where the roles of specific molecules such as glucagon could be studied in a more controlled manner.

## Supporting Information

S1 File Table A. Gene ID, GenBank accession number, hybridization position, sequence and amplicon size of primers for Bos taurus used to analyze gene expression by qPCR.Table B. qPCR performance among the genes measured in healthy and ketotic transition dairy cows.Table C. qPCR performance among the genes measured in control and dietary L-carnitine-supplemented transition dairy cows.Table D. qPCR performance among the genes measured in transition dairy cows consuming control or higher energy diets prepartum with or without a postpartal intramammary lipopolysaccharide (LPS) challenge. (DOCX)
