# Supplementary material for: Alterations in Hepatic FGF21, Co-Regulated Genes, and Upstream Metabolic Genes in Response to Nutrition, Ketosis and Inflammation in Peripartal Holstein Cows
Source: PLoS One. 2015 Oct 9;10(10):e0139963. doi: 10.1371/journal.pone.0139963 (PMC4599736; doi:10.1371/journal.pone.0139963)
Supplement: S1 File — (DOCX) [file pone.0139963.s001.docx]

## **SUPPORTING INFORMATION**

## **S1 File**

**Table A**. Gene ID, GenBank accession number, hybridization position, sequence and amplicon size of primers for Bos taurus used to analyze gene expression by qPCR.

| Gene ID | Accession # | Symbol | Primers^1^ | Primers (5’-3’) | bp^2^ | Source |
| --- | --- | --- | --- | --- | --- | --- |
| 785576 | XM_002695200.2 | *FGF21* | F.514  R.639 | CAGAGCCCCGAAAGTCTCTTG  AAAGTGCAGCGATCCGTACAG | 125 | ([Akbar et al., 2014](#_ENREF_1)) |
| 514925 | [XR_027346.2](http://www.ncbi.nlm.nih.gov/entrez/query.fcgi?cmd=Retrieve&db=Nucleotide&list_uids=194667819&dopt=GenBank&RID=TXBDMBJP014&log$=nucltop&blast_rank=1) | *KLB* | F.2833  R.2932 | TTCACACCCGATTTCAAAGCTA  GGCTGCATCGAGGACTACTGT | 100 | ([Khan et al., 2014](#_ENREF_2)) |
| 281992 | [NM_001034036.1](http://www.ncbi.nlm.nih.gov/nucleotide/77404270?report=genbank&log$=nucltop&blast_rank=2&RID=DUZ8T2SE01N) | *PPARA* | F.729  R.830 | CATAACGCGATTCGTTTTGGA  CGCGGTTTCGGAATCTTCT | 102 | ([Akbar et al., 2014](#_ENREF_1)) |
| 506812 | [FJ415874.1](http://www.ncbi.nlm.nih.gov/nucleotide/212675313?report=genbank&log$=nucltop&blast_rank=2&RID=E83NR90001S) | *CPT1A* | F.141  R.240 | TCGCGATGGACTTGCTGTATA  CGGTCCAGTTTGCGTCTGTA | 100 | ([Akbar et al., 2014](#_ENREF_1)) |
| 509963 | [NM_001046043.2](http://www.ncbi.nlm.nih.gov/entrez/query.fcgi?cmd=Retrieve&db=Nucleotide&list_uids=114326295&dopt=GenBank&RID=ZUSZNNTW012&log$=nucltop&blast_rank=2) | *ANGPTL4* | F.28  R.136 | AGGAAGAGGCTGCCCAAGAT  CCCTCTCTCCCTCTTCAAACAG | 109 | ([Loor et al., 2007](#_ENREF_3)) |
| 281209 | NM_001075240.1 | *NFIL3* | F.958  R.1057 | CAGGTCAACCGATCCTCCAGT  TGGGAACCTGCTGCTCATCT | 100 | ([Akbar et al., 2014](#_ENREF_1)) |
| 100300400 | [XM_010806118](http://www.ncbi.nlm.nih.gov/nuccore/XM_010806118) | *CLOCK* | F.608  R.709 | GGATCCATGCTTCCTGGTAATG  TGACTGTGCAGTGATTTCTTTATGTT | 101 | ([Wang et al., 2015](#_ENREF_4)) |
| 530705 | [XM_005215980](http://www.ncbi.nlm.nih.gov/nuccore/XM_005215980) | *ARNTL* | F.695  R.812 | GCGTCGGGATAAAATGAACAG  CATGTGCTGAACAGCCATCCT | 117 | ([Wang et al., 2015](#_ENREF_4)) |
| 280991 | [NM_173986.2](http://www.ncbi.nlm.nih.gov/entrez/query.fcgi?cmd=Retrieve&db=Nucleotide&list_uids=31343104&dopt=GenBank&RID=U766RPN5012&log$=nucltop&blast_rank=1) | *AKT1* | F.864  R.963 | GGATTACCTGCACTCGGAAAAG  TCCGAAGTCGGTGATCTTGAT | 100 | ([Akbar et al., 2014](#_ENREF_1)) |

^1^Primer direction (F – forward; R – reverse) and hybridization position on the sequence.

^2^Amplicon size in base pair (bp).

**Table B.** qPCR performance among the genes measured in healthy and ketotic transition dairy cows.

| Gene | Median Ct^1^ | Median ∆Ct^2^ | Slope^3^ | (R^2^)^4^ | Efficiency^5^ |
| --- | --- | --- | --- | --- | --- |
| *FGF21* | 25.33 | -5.55 | -3.15 | 0.94 | 2.08 |
| *KLB* | 24.13 | -4.36 | -3.3 | 0.91 | 2.01 |
| *NFIL3* | 25.07 | -5.29 | -3.41 | 0.98 | 1.96 |
| *CLOCK* | 25.97 | -2.56 | -3.25 | 0.99 | 2.03 |
| *ARNTL* | 26.98 | -5.72 | -3.38 | 0.94 | 1.98 |
| *AKT1* | 26.94 | -3.53 | -3.35 | 0.99 | 1.99 |

^1^The median is calculated considering all time points and all cows.

^2^The median of ∆Ct is calculated as [Ct gene – geometrical mean of Ct internal controls] for each time point and each cow.

^3^Slope of the standard curve.

^4^R^2^ stands for the coefficient of determination of the standard curve.

^5^Efficiency is calculated as [10^(-1 / Slope)^].

**Table C.** qPCR performance among the genes measured in control and dietary L-carnitine-supplemented transition dairy cows.

| Gene | Median Ct^1^ | Median ∆Ct^2^ | Slope^3^ | (R^2^)^4^ | Efficiency^5^ |
| --- | --- | --- | --- | --- | --- |
| *FGF21* | 26.53 | -5.72 | -3.43 | 0.99 | 1.96 |
| *KLB* | 22.99 | -2.18 | -3.22 | 0.99 | 2.04 |
| *PPARA* | 21.64 | -0.83 | -3.26 | 0.99 | 2.03 |
| *CPT1A* | 18.83 | 1.98 | -3.31 | 0.99 | 2.01 |
| *ANGPTL4* | 24.19 | -3.09 | -3.39 | 0.99 | 1.97 |
| *NFIL3* | 25.14 | -0.83 | -3.72 | 0.98 | 1.86 |
| *CLOCK* | 27.33 | -3.36 | -3.20 | 0.96 | 2.05 |
| *ARNTL* | 31.24 | -7.27 | -3.34 | 0.98 | 1.99 |
| *AKT1* | 28.07 | -4.10 | -3.12 | 0.91 | 2.09 |

^1^The median is calculated considering all time points and all cows.

^2^The median of ∆Ct is calculated as [Ct gene – geometrical mean of Ct internal controls] for each time point and each cow.

^3^Slope of the standard curve.

^4^R^2^ stands for the coefficient of determination of the standard curve.

^5^Efficiency is calculated as [10^(-1 / Slope)^].

**Table D.** qPCR performance among the genes measured in transition dairy cows consuming control or higher energy diets prepartum with or without a postpartal intramammary lipopolysaccharide (LPS) challenge.

| Gene | Median Ct^1^ | Median ∆Ct^2^ | Slope^3^ | (R^2^)^4^ | Efficiency^5^ |
| --- | --- | --- | --- | --- | --- |
| *FGF21* | 21.23 | 0.83 | -3.35 | 0.98 | 1.99 |
| *KLB* | 28.12 | 7.72 | -3.03 | 0.99 | 2.13 |
| *PPARA* | 21.61 | 1.21 | -3.06 | 0.99 | 2.12 |
| *CPT1A* | 20.55 | 0.15 | -3.15 | 0.99 | 2.07 |
| *ANGPTL4* | 23.14 | 2.74 | -3.41 | 0.97 | 1.96 |
| *NFIL3* | 23.27 | 2.87 | -3.27 | 0.97 | 2.02 |
| *CLOCK* | 27.33 | -3.36 | -3.20 | 0.96 | 2.05 |
| *ARNTL* | 29.08 | 8.68 | -3.20 | 0.99 | 2.05 |
| *AKT1* | 22.90 | 2.50 | -3.52 | 0.99 | 1.92 |

^1^The median is calculated considering all time points and all cows.

^2^The median of ∆Ct is calculated as [Ct gene – geometrical mean of Ct internal controls] for each time point and each cow.

^3^Slope of the standard curve.

^4^R^2^ stands for the coefficient of determination of the standard curve.

^5^Efficiency is calculated as [10^(-1 / Slope)^].

**References**

Akbar, H., F. C. Cardoso, S. Meier, C. Burke, S. McDougall, M. Mitchell, C. Walker, S. L. Rodriguez-Zas, R. E. Everts, H. A. Lewin, J. R. Roche, and J. J. Loor. 2014. Postpartal subclinical endometritis alters transcriptome profiles in liver and adipose tissue of dairy cows. Bioinformatics and biology insights 8:45-63.

Khan, M. J., C. B. Jacometo, D. E. Graugnard, M. N. Correa, E. Schmitt, F. Cardoso, and J. J. Loor. 2014. Overfeeding Dairy Cattle During Late-Pregnancy Alters Hepatic PPARalpha-Regulated Pathways Including Hepatokines: Impact on Metabolism and Peripheral Insulin Sensitivity. Gene regulation and systems biology 8:97-111.

Loor, J. J., R. E. Everts, M. Bionaz, H. M. Dann, D. E. Morin, R. Oliveira, S. L. Rodriguez-Zas, J. K. Drackley, and H. A. Lewin. 2007. Nutrition-induced ketosis alters metabolic and signaling gene networks in liver of periparturient dairy cows. Physiological genomics 32(1):105-116.

Wang, M., Z. Zhou, M. J. Khan, J. Gao, and J. J. Loor. 2015. Clock circadian regulator (CLOCK) gene network expression patterns in bovine adipose, liver, and mammary gland at 3 time points during the transition from pregnancy into lactation. J Dairy Sci.
